# Estrogen Receptor-Alpha 36 Mediates Mitogenic Antiestrogen Signaling in ER-Negative Breast Cancer Cells

**DOI:** 10.1371/journal.pone.0030174

**Published:** 2012-01-19

**Authors:** XinTian Zhang, Ling Ding, LianGuo Kang, Zhao-Yi Wang

**Affiliations:** 1 Department of Medical Microbiology and Immunology, Creighton University Medical School, Omaha, Nebraska, United States of America; 2 Department of Oncology, Second Affiliated Hospital, Zhejiang University School of Medicine, Hangzhou, Zhejiang, People's Republic of China; Institute of Molecular and Cell Biology, Singapore

## Abstract

It is prevailingly thought that the antiestrogens tamoxifen and ICI 182, 780 are competitive antagonists of the estrogen-binding site of the estrogen receptor-alpha (ER-α). However, a plethora of evidence demonstrated both antiestrogens exhibit agonist activities in different systems such as activation of the membrane-initiated signaling pathways. The mechanisms by which antiestrogens mediate estrogen-like activities have not been fully established. Previously, a variant of ER-α, EP–α36, has been cloned and showed to mediate membrane-initiated estrogen and antiestrogen signaling in cells only expressing ER-α36. Here, we investigated the molecular mechanisms underlying the antiestrogen signaling in ER-negative breast cancer MDA-MB-231 and MDA-MB-436 cells that express high levels of endogenous ER-α36. We found that the effects of both 4-hydoxytamoxifen (4-OHT) and ICI 182, 780 (ICI) exhibited a non-monotonic, or biphasic dose response curve; antiestrogens at low concentrations, elicited a mitogenic signaling pathway to stimulate cell proliferation while at high concentrations, antiestrogens inhibited cell growth. Antiestrogens at l nM induced the phosphorylation of the Src-Y416 residue, an event to activate Src, while at 5 µM induced Src-Y527 phosphorylation that inactivates Src. Antiestrogens at 1 nM also induced phosphorylation of the MAPK/ERK and activated the Cyclin D1 promoter activity through the Src/EGFR/STAT5 pathways but not at 5 µM. Knock-down of ER-α36 abrogated the biphasic antiestrogen signaling in these cells. Our results thus indicated that ER-α36 mediates biphasic antiestrogen signaling in the ER-negative breast cancer cells and Src functions as a switch of antiestrogen signaling dependent on concentrations of antiestrogens through the EGFR/STAT5 pathway.

## Introduction

The diverse physiological functions of estrogens are mediated by estrogen receptors ER-α and ER-β, both of which are ligand-activated transcription factors that stimulate target gene transcription [1]. Estrogen-induced transcription regulation has been prevailingly thought as the only mechanism of estrogen action. However, it became apparent now that not all of the physiological effects mediated by estrogens are accomplished through a direct effect on gene transcription. Another signaling pathway (also known as a ‘non-classic,’ ‘non-genomic’ or ‘membrane-initiated’ signaling pathway) exists that involves cytoplasmic signaling proteins, growth factor receptors and components of other membrane-initiated signaling pathways [2], [3].

Since mitogenic estrogen signaling plays a pivotal role in development and progression of ER-positive breast cancer, treatment with antiestrogens such as tamoxifen (TAM) has become a first-line therapy for advanced ER-positive breast cancer. However, laboratory and clinical evidence indicated that TAM and its metabolites such as 4-hydroxytamoxifen (4-OHT) have mixed agonist/antagonist or estrogenic/anti-estrogenic actions depending on cell and tissue context, and the agonist activity of tamoxifen may contribute to tamoxifen resistance observed in almost all patients treated with tamoxifen [4], [5], [6]. As a consequence, a more potent and “pure” antiestrogen, ICI 182, 780 (Fulvestrant, Faslodex) has been developed [7].

TAM and 4-OHT are thought to function as antagonists by competing with 17-β-estradiol (E2β) and other estrogens for binding to ERs. Further structural studies revealed that TAM induces an ER-α conformation that does not recruit coactivators to trans-activate target genes but recruits co-repressors [8], suggesting that TAM- and 4-OHT-bounded ER-α is unable to effectively activate genes involved in cell growth and breast cancer development. On the other hand, ICI 182, 780, a ‘pure’ antiestrogen, works in a different mechanism. ICI 182, 780 binds to ERs, impairs receptor dimerization and inhibits nuclear localization of receptor [9], [10]. Furthermore, ICI 182, 780 also accelerates degradation of the ER-α protein without a reduction of ER-α mRNA [10], [11]. Thus, ICI 182, 780 binds ER-α and accelerates degradation of ER-α protein, resulting in a complete inhibition of estrogen signaling mediated by ER-α.

Although ICI 182, 780 has been depicted as a non-agonist or ‘full’ or ‘pure’ antiestrogen, a number of laboratories reported estrogenic agonist activities of ICI 182, 780 in different systems. Estrogenic agonist activity of ICI 182, 780 has been reported in hippocampal neurons and in bone cells where ICI 182, 780 promoted bone growth [12], [13]. Agonist-like activities of ICI 182, 780 have also been reported in human breast cancer cells [14], sheep uterus [15] and yeast [16]. The molecular mechanisms by which ICI 182, 780 acts as an estrogenic agonist have never been elucidated. Studies from several laboratories suggested that a membrane-associated estrogen-binding receptor mediates the agonist actions of ICI 182, 780 in neurons [17], [18], [19], [20].

Previously, we identified and cloned a 36-kDa variant of ER-α, ER-α36 [21]. ER-α36 lacks both transcription activation domains AF-1 and AF-2 of the 66 kDa ER-α (ER-α66), consistent with the fact that ER-α36 has no intrinsic transcriptional activity [21], [22] ER-α36 transcripts are generated from a promoter located in the first intron of the ER-α66 gene [23], indicating that ER-α36 expression is regulated differently from ER-α66. Indeed, ER-α36 is expressed in specimens from ER-negative patients and ER-negative breast cancer cells that lack ER-α66 expression [24], [25], [26]. ER-α36 is mainly expressed on the plasma membrane and mediates membrane-initiated estrogen signaling [22], [27]. Antiestrogens such as TAM and ICI 182, 780 at 10 nM induced phosphorylation of the MAPK/ERK in HEK/293 cells expressing recombinant ER-α36 [22]. ER-α36 also mediates agonist activity of tamoxifen in endometrial cancer cells [28]. These results suggested that ER-α36-mediated non-genomic signaling pathway is involved in agonist activities of antiestrogens.

Recently, we reported that ER-α36 mediated mitogenic estrogen signaling in ER-negative breast cancer cells such as MDA-MB-231 and MDA-MB-436 cells that lack expression of ER-α66 but highly express ER-α36 [29]. To exclude the involvement of ER-α66, we used these cells to study the effects and the underlying mechanisms of pharmacological high concentrations and clinical relevant low concentrations of antiestrogens. In addition, although MDA-MB-231 cells express the full-length ER-β, MDA-MB-436 cells express undetectable levels of full-length ER-β [29], which will then exclude the possible involvement of ER-β.

In the current study, we examined the agonist activities of antiestrogens ICI 182, 780 and 4-OHT in the ER-negative breast cancer MDA-MB-231 and MDA-MB-436 cells and found that the ER-negative breast cancer cells exhibited biphasic growth response curves in response to these antiestrogens. We also found that ER-α36-mediated Src/EGFR/STAT5 signaling pathway plays an important role in the biphasic antiestrogen signaling.

## Results

### Antiestrogens stimulates proliferation of ER-negative breast cancer cells

**Figure 1:**
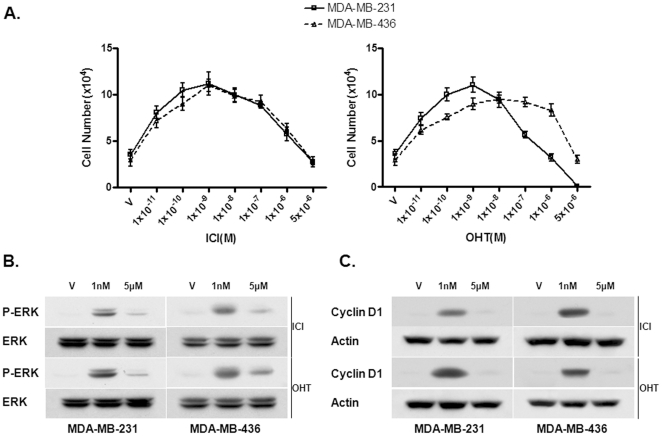
ER-negative breast cancer cells exhibit biphasic antistrogen signaling. (A). The effects of 4-OHT and ICI 182, 780 on the proliferation rate of MDA-MB-231 and MDA-MB-436 cells. Cells maintained for three days in phenol red-free DMEM plus 2.5% dextran-charcoal-stripped fetal calf serum were treated with indicated concentrations of 4-OHT, ICI or ethanol vehicle as a control. The cell numbers were determined using an automatic cell counter after 12 days. Five dishes were used for each concentration and experiments were repeated more than three times. The mean cell number ± SE are shown. (B). The dose-dependent phosphorylation pattern of the MAPK/ERK1/2 in MDA-MB-231 and MDA-MB-436 cells treated with different concentrations of antiestrogens. Starved cells were treated with indicated doses of 4-OHT or ICI 182, 780 (ICI) for 10 min. Western blot analysis was performed to assess induction of ERK1/2 phosphorylation. The experiment was repeated more than three times. The representative results are shown. (C). The dose dependent induction Cyclin D1 by antiestrogens in MDA-MB-231 and MDA-MB-436 cells. The experiment was repeated more than three times. The representative results are shown.

To test if antiestrogens such as ICI 182, 780 (ICI) and 4-OHT act as agonists in the ER-negative breast cancer cells, the growth rate of each cell line was determined by counting the number of cells cultured in different concentrations of ICI and 4-OHT. As shown in Figure 1A, the ER-negative breast cancer cells treated with low concentrations (<1 nM) antiestrogens exhibited an increased growth rate compared with cells treated with vehicle. The dose-response curves of these cells to antiestrogens exhibited a non-monotonic or biphasic pattern; increasing concentrations of antiestrogens that initially stimulated cell growth but inhibited cell growth at higher concentrations (Figure 1A). Our data indicated that antiestrogens induced proliferation of ER-negative breast cancer cells in a biphasic pattern.

### Antiestrogens induces biphasic activation of the MAPK/ERK and Cyclin D1 expression in ER-negative breast cancer cells

To determine whether antiestrogens induced phosphorylation of the MAPK/ERK1/2, a typical non-genomic estrogen-signaling event, in these two cell lines, we treated cells with ICI and 4-OHT at different concentrations (1 nM and 5 µM) for 10 min. These concentrations were chosen to reflect physiological estrogen concentration and pharmacological antiestrogens concentration, respectively. Western blot analysis with a phospho-specific ERK1/2 antibody was performed to assess the phosphorylation levels of the ERK1/2. As shown in Figure 1B, we found that both ICI and 4-OHT were able to induce the activation of the MAPK/ERK at a low concentration (1 nM) in both cell lines. However, the activation of the MAPK/ERK was not observed in cells treated with a high concentration (5 µM) of ICI and 4-OHT (Figure 1B), consistent with the biphasic pattern of the dose-response curves of these cells to ICI and 4-OHT. To determine whether high concentrations of antiestrogens failed to activate the MAPK/ERK or inhibited the ERK activation, we examined the effects of high concentrations of ICI and 4-OHT on the ERK activation induced by EGF. We found that antiestrogens failed to inhibit ERK activation induced by EGF (data not shown), suggesting that high concentrations of antiestrogens may fail to activate the MAPK/ERK.

It is well known that induction of the growth-promoting gene Cyclin D1 by estrogen contributes to estrogen-stimulated proliferation of ER-positive breast cancer cells. Previously, we reported that E2β was also able to induce expression of c-Myc and Cyclin D1 in the ER-negative breast cancer cells [22]. To assess whether antiestrogens were also able to induce expression of Cyclin D1, we treated cells with two concentrations (1 nM or 5 µM) of antiestrogens for six hours, and Western blot analysis was performed to examine Cyclin D1 expression. We found that at 1 nM, both ICI and 4-OHT up-regulated expression levels of Cyclin D1 (Figure 1C) whereas at 5 µM, both antiestrogens failed to induce Cyclin D1 expression (Figure 1C). Thus, antiestrogens elicited a biphasic induction of Cyclin D1 expression in these ER-negative breast cancer cells.

### Src/EGFR/STAT5 are involved in biphasic antiestrogen signaling in ER-negative breast cancer cells

**Figure 2:**
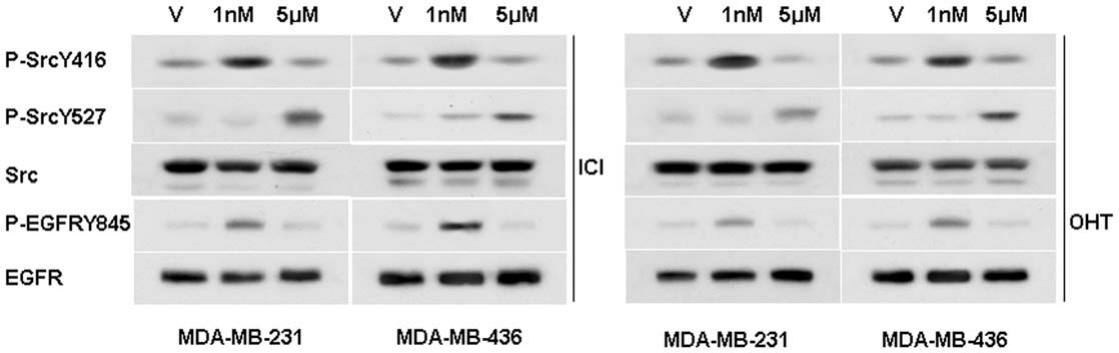
Different concentrations of antiestrogens induce Src phosphorylation at distinct residues. Western blot analysis of the effects of different concentrations of antiestrogens on the phosphorylation levels of EGFR-Y845, Src-Y416 and Src-Y527 in MDA-MB-231 and MDA-MB-436 cells.

Recently, we reported that E2β induced phosphorylation of Src-Tyr-416 and activated Src activity, which then induced phosphorylation of EGFR-Tyr-845 in these ER-negative breast cancer cells [22]. We then examined the phosphorylation status of Src-Tyr-416 and EGFR-Tyr-845 in the cells treated with different concentrations of antiestrogens. Figure 2 shows that in both cell lines, 1 nM of ICI and 4-OHT elicited phosphorylation of Src-Tyr-416 and EGFR-Tyr-845 while failed to do so at 5 µM. Intriguingly, 5 µM of ICI and 4-OHT strongly induced phosphorylation of Src-Tyr-527, an event associated with inactivation of Src activity, which was not observed in the cells treated with 1 nM of antiestrogens. These results suggested that antiestrogens at low concentrations induced phosphorylation of Src-Y-416 and activated Src whereas at high concentrations, antiestrogens induced Src-Y-527 phosphorylation and inactivated Src activity.

**Figure 3:**
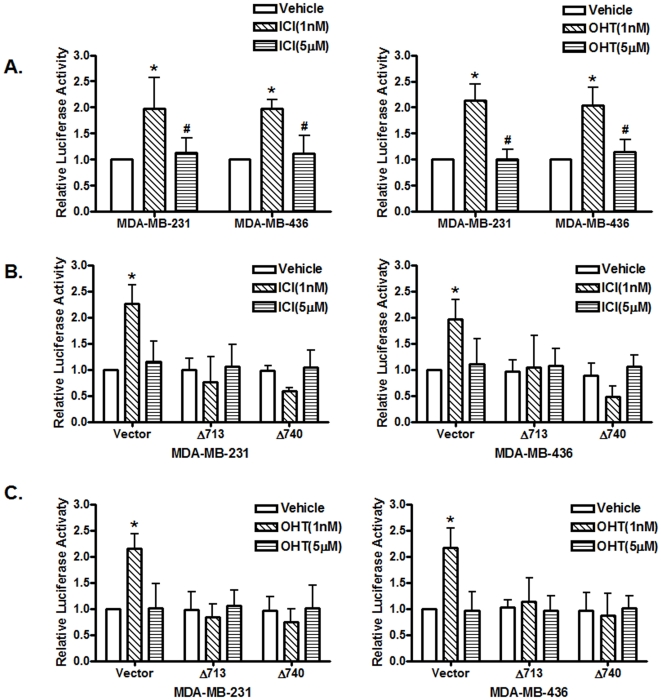
Antiestrogens induce biphasic STAT5 activities in ER-negative breast cancer cells. (A). ER-negative breast cancer cells were transfected with the luciferase reported plasmid 4XM67 TATA-TK-Luc that containing four copies of STAT-binding sites upstream of the minimal TK promoter. Transfected cells were treated with vehicle (ethanol), 1 nM or 5 µM of 4-OHT or ICI 182, 780. The luciferase activities were assayed and normalized using a cytomegalovirus-driven Renilla luciferase plasmid. Columns: means of the relative luciferase activity from four independent experiments. Luciferase activity in transfected cells treated with vehicle is arbitrarily set as 1.0; bars, SE. *, p<0.05, for cells treated with vehicle (V) vs 1 nM of antiestrogens. #, p<0.05, for cells treated with 5 µM vs 1 nM of antiestrogens. (B&C). Cells were transfected with the 4XM67 TATA-TK-Luc reporter together with an empty expression vector (vector) and the expression vectors of two dominant-negative STAT5a mutants carrying truncations at their C-terminal (STAT5aΔ713 and STAT5aΔ740) before treated with vehicle (ethanol), 1 nM or 5 µM of antiestrogens. Columns: means of the relative luciferase activity from three independent experiments. Luciferase activity of cells co-transfected with an empty expression vector and treated with vehicle is arbitrarily set as 1.0; bars, SE. *, p<0.05, for cells treated with vehicle (V) vs 1 nM of antiestrogens.

It was reported that signal transducer and activator of transcription 5 (STAT5), Src and EGFR play important roles in estrogen-stimulated proliferation of ER-positive breast cancer cells [30]; estrogen-induced Src activation and Src-dependent phosphorylation of EGFR-Tyr-845 recruit STAT5 as a downstream effector of phosphorylated EGFR-Tyr-845 [30]. To examine whether STAT5 is involved in the observed biphasic antiestrogen signaling, we transfected MDA-MB-231 and MDA-MB-436 cells with the 4 X M67 pTATA-TK-luciferase reporter plasmid that contains four copies of STAT-binding site and treated with antiestrogens at 1 nM and 5 µM. We found that 1 nM of antiestrogens activated the promoter activity of the reporter plasmid while 5 µM of antiestrogens failed to do so (Figure 3A), suggesting that antiestrogens at low concentrations were able to activate STAT-mediated transcription. To confirm the involvement of STAT5, we included two dominant-negative mutants of STAT5a (STAT5aΔ713 and STAT5aΔ740) that inhibit transcription activation mediated by STAT5a/b [31]. We found that both dominant-negative mutants of STAT5a potently inhibited 1 nM of both ICI and 4-OHT induced promoter activity of the 4 X M67 pTATA-TK-luciferase reporter plasmid (Figure 3B & C), indicating that STAT5 is involved in the biphasic antiestrogen signaling.

### Src is involved in biphasic Cyclin D1 expression induced by different concentrations of antiestrogens

**Figure 4:**
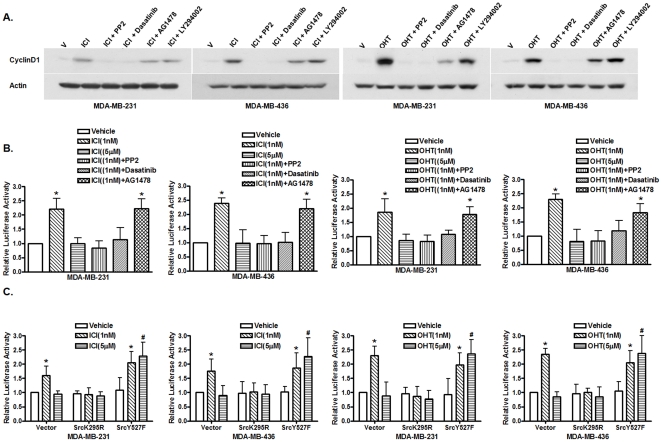
Src is involved in antiestrogen-induced Cyclin D1 expression. (A). Western blot analysis of Cyclin D1 expression in MDA-MB-231 and -436 cells. Cells were treated with vehicle (ethanol) and antiestrogens alone or together with the Src inhibitors PP2 and dasatinib, the EGFR inhibitor AG1478 and PI3K inhibitor LY294002. Cell lysates were analyzed with anti-Cyclin D1 antibody and anti-Acin antibody was used to ensure equal loading. The experiment was repeated three times, and the representative results are shown. (B). Src inhibitors inhibit antiestrogen-induced Cyclin D1 promoter activity. ER-negative breast cancer cells were transfected with the luciferase reported plasmid Cyclin D1 pl-963 that containing a luciferase gene driven by the Cyclin D1 promoter. Transfected cells were treated with vehicle (ethanol), 1 nM or 5 µM of antiestrogens, and 1 nM of antiestrogens together with different inhibitors. The luciferase activities were assayed and normalized using a cytomegalovirus promoter-driven Renilla luciferase plasmid. Columns: means of the relative luciferase activity in cells treated with vehicle that is arbitrarily set as 1.0 from four independent experiments; bars, SE. *, p<0.05, for cells treated with vehicle (V) vs 1 nM of antiestrogens, or vehicle (V) vs 1 nM of antiestrogens plus AG1478. (C). The involvement of Src in antiestrogen-induced Cyclin D1 promoter activity. Cells were transfected with the luciferase reported plasmid Cyclin D1 pl-963 together with an empty expression vector or Src mutants, a dominant-negative mutant (SrcK295R) and a constitutively active mutant (SrcY527F). Transfected cells were treated with vehicle (ethanol), 1 nM or 5 µM of antiestrogens. The luciferase activities were assayed and normalized using a cytomegalovirus-driven Renilla luciferase plasmid. Columns: means of the relative luciferase activity from four independent experiments. Luciferase activity in transfected cells treated with vehicle is arbitrarily set as 1.0; bars, SE. *, p<0.05, for cells treated with vehicle (V) vs 1 nM of antiestrogens. #, p<0.05, for cells treated with vehicle (V) vs 5 µM of antiestrogens.

In the experiments described above, we observed that the cells treated with different concentrations of antiestrogens also exhibited biphasic patterns of Cyclin D1 expression. We decided to examine whether the Src signaling pathway is involved in the induction of Cyclin D1 expression by low concentrations of antiestrogens. We first tested if the Src inhibitors PP2 and dasatinib were able to inhibit Cyclin D1 induction by 1 nM of antiestrogens. Cells were treated with 1 nM of either ICI or 4-OHT and together with the Src inhibitors PP2 and dasatinib, the EGFR inhibitor AG1478 or the PI3K inhibitor LY294002, and Western blot analysis was performed to examine Cyclin D1 expression. Figure 4A shows that 1 nM antiestrogen-induced Cyclin D1 expression was blocked by the Src inhibitors but not by AG1478 and LY294002, suggesting that Src is involved in Cyclin D1 expression induced by low concentrations of antiestrogens in these ER-negative breast cancer cells. To confirm Src function in Cyclin D1 induction by antiestrogens, we transiently transfected both cell lines with a human Cyclin D1 promoter-luciferase construct and then treated transfected cells with 1 nM or 5 µM antiestrogens. We found that 1 nM of both antiestrogens was able to induce Cyclin D1 promoter activity whereas at 5 µM, both antiestrogens failed to induce Cyclin D1 promoter activity (Figure 4B), indicating the biphasic effects of antiestrogens on induction of Cyclin D1 expression is through regulation of its promoter activity. The Cyclin D1 promoter activity induced by 1 nM of antiestrogens was inhibited by the Src inhibitors PP2 and dasatinib but not by AG1478 (Figure 4B). To further confirm the involvement of Src in the antiestrogen-induced Cyclin D1 expression, these ER-negative breast cancer cells were transiently co-transfected with the Cyclin D1 promoter reporter plasmid and pCMV5/SrcK295M, a dominant-negative mutant of Src, or pCMV5/SrcY527F, a constitutively active mutant of Src, respectively. We found that co-transfection of the dominant-negative mutant of Src abrogated the Cyclin D1 promoter activity induced by 1 nM estrogen while had no effects in cells treated with 5 µM antiestrogens (Figure 4C). On the contrary, the constitutively active mutant of Src (SrcY527F) released the Cyclin D1 promoter activity suppressed by 5 µM antiestrogens (Figure 4C). These results indicated Src plays an integral role in biphasic response of Cyclin D1 to different concentrations of antiestrogens.

### STAT5 is involved in antiestrogen induced Cyclin D1 promoter activity

**Figure 5:**
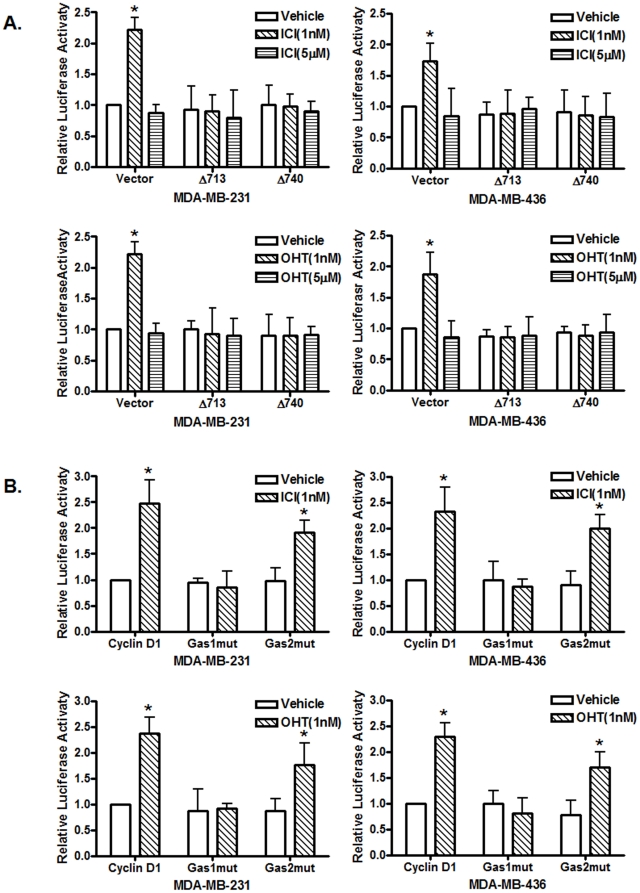
STAT5 is involved in antiestrogen-induced Cyclin D1 promoter activity. (A). The involvement of STAT5 in antiestrogens-induced Cyclin D1 promoter activity. Cells were transfected with the luciferase reported plasmid Cyclin D1 pl-963 together with an empty expression vector or two dominant-negative STAT5a mutants, STAT5aΔ713 and STAT5aΔ740, respectively. Transfected cells were treated with vehicle (ethanol), 1 nM or 5 µM of antiestrogens. Columns: means of the relative luciferase activity from four independent experiments. Luciferase activity in the cells transfected with an empty expression vector and treated with vehicle is arbitrarily set as 1.0; bars, SE. *, p<0.05, for cells treated with vehicle (V) vs 1 nM of antiestrogens. (B). The GAS1 is involved in induction of the Cyclin D1 promoter activity by antiestrogens. Cells were transiently transfected with either the wild-type Cyclin D1 promoter (CycD1) or the same promoter construct containing mutated GAS1 (GAS1mut) or GAS2 (GAS2mut) sequence, respectively. Transfected cells were treated with vehicle or 1 nM of antiestrogens, and the luciferase activity was presented relative to the wild-type Cyclin D1 promoter-transfected cells treated with vehicle that is arbitrarily set as 1.0. *, p<0.05, for cells treated with vehicle (V) vs 1 nM of antiestrogens.

Previously, it was reported that prolactin induces Cyclin D1 promoter activity through activation of STAT proteins and their interaction with the consensus gamma-interferon-activation sites (GAS) located in the Cyclin D1 promoter [32]. We decided to examine whether antiestrogens function the same as prolactin in these ER-negative breast cancer cells. Two dominant-negative mutants of STAT5a were co-transfected with the Cyclin D1 promoter reporter plasmid, and the transfected cells were treated with 1 nM of ICI or 4-OHT. We found that inclusion of the two mutants of STAT5a strongly suppressed the Cyclin D1 promoter activity induced by 1 nM of antiestrogens (Figure 5A), indicating that 1 nM of antiestrogens induced the Cyclin D1 promoter activity through STAT5 in ER-negative breast cancer cells.

In human cyclin D1 promoter, there are two GAS consensus sequences at −457 (GAS1) and −224 (GAS2) (relative to the transcription initiation site) that have been previously shown to be sites for STAT protein binding induced by prolactin [32]. To assess involvement of the two GAS sequences in antiestrogen-induced Cyclin D1 promoter activity, we transiently transfected these ER-negative breast cancer cells with two mutants of the Cyclin D1 promoter/reporter constructs, GAS1mut and GAS2mut that mutated the two GAS sequences respectively to prevent STAT protein binding. The Cyclin D1 promoter containing the GAS1 mutation failed to respond to 1 nM of ICI or 4-OHT while GAS2 mutant retained the ability to response to 1 nM of both antiestrogens (Figure 5B), indicating that the STAT-binding GAS1 site is involved in the increase of Cyclin D1 promoter activity induced by low-concentrations of antiestrogens.

### ER-α36 mediates mitogenic antiestrogen signaling in ER-negative breast cancer cells

**Figure 6:**
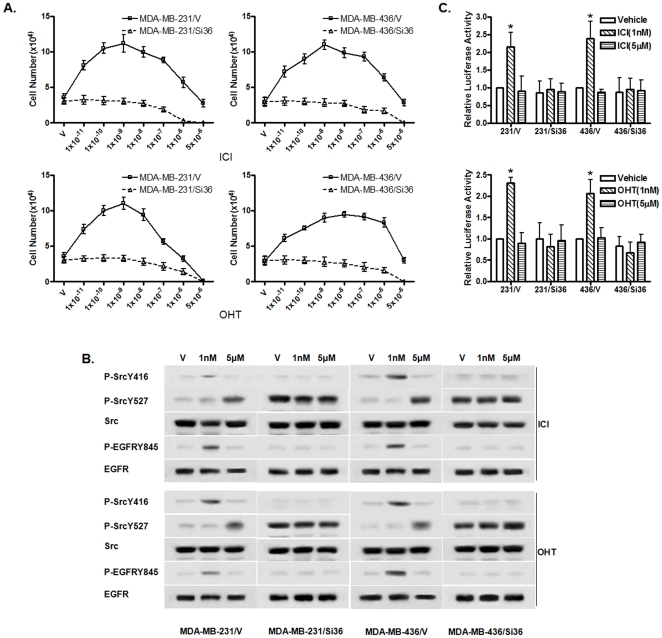
ER-α36 mediates biphasic antiestrogen signaling in ER-negative breast cancer cells. (A). The effects of antiestrogens on the proliferation rate of MDA-MB-231 and MDA-MB-436 cells with or without ER-α36 expression knocked-down. Cells maintained for three days in phenol red-free DMEM plus 2.5% dextran-charcoal-stripped fetal calf serum were treated with indicated concentrations of antiestrogens or ethanol vehicle as a control. The cell numbers were determined using an automatic cell counter after 12 days. Five dishes were used for each concentration and experiments were repeated three times. The mean cell number ± SE are shown. (B). Western blot analysis of the effects of 1 nM or 5 µM of antiestrogens on the phosphorylation levels of the SrcY416, SrcY527 and EGFRY845 in MDA-MB-231 and MDA-MB-436 cells. (C). Cells were transfected with the luciferase reporter plasmid driven by the wild-type Cyclin D1 promoter and transfected cells were treated with vehicle (ethanol), 1 nM or 5 µM of antiestrogens. Columns: means of the relative luciferase activity in transfected cells treated with vehicle that is arbitrarily set as 1.0 from three independent experiments; bars, SE. *, p<0.05, for cells treated with vehicle (V) vs 1 nM of antiestrogens.

Previously, we reported that ER-α36 mediates mitogenic estrogen signaling in ER-negative breast cancer MDA-MB-231 and MDA-MB-436 cells using shRNA method [29]. To determine the involvement of ER-α36 in the antiestrogen signaling of these breast cancer cells, we tested antiestrogen signaling in the cell lines derived from MDA-MB-231 and MDA-MB-436 that carrying knocked-down levels of ER-(36 expression by the shRNA method. Cells derived from both cell lines with ER-(36 expression knocked-down by shRNA failed to increase cell proliferation in response to low-concentrations of antiestrogens (Figure 6A), suggesting that ER-(36 mediates mitogenic antiestrogen signaling in these ER-negative breast cancer cells. However, at 5 µM, both antiestrogens potently inhibited proliferation of MDA-MB-231 and MDA-MB-436 cells with knocked-down levels of ER-α36 expression (Figure 6A).

We found that 1 nM antiestrogens failed to induce Src-Tyr-416 and EGFR-Tyr-845 phosphorylation in MDA-MB-231 and MDA-MB-436 cells with knocked-down level of ER-α36 expression (Figure 6B). However, the basal levels of Src-Tyr-527 phosphorylation were dramatically increased in MDA-MB-231 and –436 cells transfected with ER-α36 shRNA expression vector compared to control cells transfected with the empty expression vector (Figure 6B), which was not further induced by 5 µM of antiestrogens (Figure 6B). We also tested whether antiestrogensare able to induce Cyclin D1 promoter activity in the cells with ER-α36 knock-down. Both antiestrogens at 1 nM failed to induce Cyclin D1 promoter activity (Figure 6C).

### Different concentrations of antiestrogens affect the association of ER-α36 and Src differently

**Figure 7:**
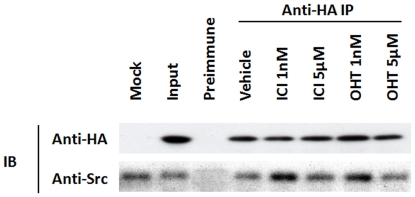
Different concentrations of antiestrogens affect the association of ER-α36 and Src differently. Co-immunoprecipitation and Western blot analysis of HA-ER-α36 and Src in MDA-MB-231 cells. Cells transiently transfected with an expression of HA-tagged ER-α36 and treated with different concentrations of antiestrogens for 10 min were lysised and the cell lysates were immunoprecipitated with pre-immune and anti-HA antibodies. The immunoprecipitates were blotted by anti-HA and anti-Src antibodies.

To elucidate the molecular mechanism by which different concentrations of antiestrogens influence Src phosphorylation in ER-negative breast cancer cells, we examined the effects of different concentrations of antiestrogens on the association of ER-α36 with Src as we reported before [29]. MDA-MB-231 cells were transiently transfected with an expression vector for HA-tagged ER-α36 and treated with different concentrations of antiestrogens for 10 min. Cell lysates were immunoprecipitated with pre-immune and anti-HA antibodies, and blotted by anti-HA and anti-Src antibodies. Figure 7 shows that at l nM, antiestrogens induced association of ER-α36 and Src, which was decreased when treated with 5 µM of antiestrogens (Figure 7). This result demonstrated that antiestrogens at 1 nM induced interaction between ER-α36 and Src but failed to do so at 5 µM.

## Discussion

Here, we used ER-negative breast cancer MDA-MB-231 and MDA-MB-436 cells as models to study the effects and the underlying mechanisms of the rapid, non-genomic antiestrogen signaling mediated by ER-α36. We found that these ER-negative breast cancer cells exhibited a biphasic growth response curve to antiestrogens ICI 182, 780 and 4-OHT; antiestrogens stimulated cell proliferation at sub-nM range while inhibited cell growth at µM range.

Antiestrogens such as tamoxifen and ICI 182, 780 are widely used for the treatment of advanced breast cancer, especially ER-positive breast cancer. It is prevailingly thought that ER-negative breast cancer is less or no responsive to antiestrogen therapy. However, it has also been reported that about 45% ER−/PR+ breast tumor patients and 10% ER−/PR− tumor patients responded to tamoxifen treatment (Reviewed in [33]), suggesting that a subset of ER-negative breast cancer still responses to antiestrogen therapy. Previously, several *in vitro* studies showed that tamoxifen and 4-OHT can cross-talk with other signaling pathways such as the p38/MAPK and the SAPK/JNK pathways, and induce apoptosis in both ER-positive breast cancer cells such as MCF7 and ER-negative cells such as MDA-MB-231 [33], [34], [35], [36]. However, we did not observe significant apoptosis in antiestrogen treated ER-negative breast cancer cells at 5 µM, the highest concentration we used, presumably because much higher concentrations of 4-OHT (≥10–20 µM) were required to induce apoptosis in ER-negative breast cancer cells [33].

It is well-known that tamoxifen and its metabolite 4-OHT act as both agonists and antagonists in a tissue specific fashion. However, the mechanisms underlying the paradox effects of tamoxfen have never been fully elucidated. Different patterns of co-regulators expression and different post-translation modifications of ER have been proposed to be involved in tissue specific agonist/antagonist properties of tamoxifen (Reviewed in [37]). We recently reported that ER-α36 mediates the agonist activity of tamoxifen by activation of the MAPK/ERK and PI3K/AKT signaling pathways in endometrial cancer cells that lack expression of ER-α66 [28]. Our current results thus suggested that the agonist/antagonist activities of tamoxifen are concentration dependent and ER-α36 is involved in the agonist/antagonist activities of tamoxifen.

ICI 182, 780 has been portrayed as a “pure” antiestrogen without any estrogenic activity [7]. Here, we found that ICI 182, 780 worked just like 4-OHT and exhibited concentration-dependent agonist/antagonist activities in cells expressing ER-α36. Recently, we reported that ICI 182, 780 failed to induce degradation of ER-α36 [38], presumably because ER-α36 has a truncated ligand-binding domain that lacks the last 4 helixes (helix 9–12) of ER-α66 [22]. The helix-12 domain is critical in protein degradation induced by ICI 182, 780 and different positioning of the helix 12 and the F domain of ER-α66 regulates functional differences between agonists and antagonists [39], [40], [41]. This may provide a molecular explanation for the failure of ICI 182, 780 to induce ER-α36 degradation and inhibited ER-α36 activity. Previously, low concentrations of ICI 182, 780 were found to stimulate the growth of tamoxifen-resistant KPL-1 human breast cancer cells [42] and to induce phosphorylation of the MAPK/ERK in neonatal rat cerebellar neurons [12]. Thus, like tamoxifen, ICI 182, 780 also has concentration-dependent agonist/antagonist activities.

It is worth noting that the earlier version of ER-α66/knockout mice that was generated by insertion of a Neo cassette into the first coding exon of the mouse ER-α gene [43] (the exon that is sipped in the generation of the transcript encoding ER-α36) retains ER-α36 expression (Elliot Sharon, personal communication). This version of ER-α66 deficient mice also retained ICI 182, 780 insensitive non-genomic estrogen-signaling in different tissues [44], [45], [46]. Our current results suggested that ER-α36 may be involved in the ICI 182, 780 resistant non-genomic estrogen signaling observed in the early version of ER-α66 deficient mice.

The involvement of Src in rapid estrogen signaling has been reported in the mouse neocortex, ER-positive breast cancer cells, and prostate cancer cells [47], [48], [49]. It has also been reported that antiestrogen tamoxifen promotes phosphorylation of the adhesion molecules, p130Cas/BCAR1, FAK and Src [50]. In the present study, we found that at 1 nM, antiestrogens induced phosphorylation of Src at Tyr-416 and the downstream MAPK/ERK1/2. Intriguingly, we found that 5 µM antiestrogenstriggered phosphorylation of Src-Tyr-527 and failed to induce phosphorylation of Src-Tyr-416 and the MAPK/ERK1/2. Src can be switched from an inactive to an active state through control of its phosphorylation state [51]. Src-Tyr-416 can be auto-phosphorylated, which activates Src by displacing the P-Tyr-416 from the binding pocket, allowing the substrate to gain access. However, phosphorylation of Tyr-527 inactivates Src through the interaction of P-Tyr-527 with a SH2 domain, which effectively folds Src up into a closed, inactive state. Our results thus demonstrated, for the first time, that phosphorylation state of Src-Y-416 and-Y-527 acts as a switch of concentration dependent agonist/antagonist activities of antiestrogens.

Previously, we reported that E2β induced the physical interaction of ER-α36 and Src, and consequently the auto-phosphorylation of Src-Y-416 in the ER-negative breast cancer cells [29]. Here, we found that ER-α36 knock-down diminished the Src-Y-416 phosphorylation induced by 1 nM of antiestrogens, indicating ER-α36 is involved in the auto-phosphorylation of Src-Y-416 induced by low-concentrations of antiestrogens. However, the cells with ER-α36 knock-down exhibited high basal levels of Src-Tyr-527 phosphorylation, which was not further induced by antiestrogens at 5 µM, indicating that abrogation of ER-α36 activity increased basal levels of Src-Tyr-527 phosphorylation and silenced Src activity, consistent with our previous findings that the ER-negative breast cancer cells with ER-α36 knock-down failed to form xenograft tumors [29]. Furthermore, in the co-immunoprecipitation assays, we found that antiestrogens at low concentration (1 nM) induced interaction between ER-α36 and Src, suggesting that like estrogen, both antiestrogens are able to induce association of ER-α36 and Src as well as auto-phosphorylation of Src. At high concentration (5 µM), antiestrogens failed to induce the interaction of ER-α36 and Src. It is possible that different concentrations of antiestrogens may trigger different conformations of ER-α36, which regulates ER-α36 accessibility for Src binding. The failure of the interaction between ER-α36 and Src may increase the basal levels of Src-Tyr-527 phosphorylation to silence Src as we observed in the cells with ER-α36 expression knocked-down.

The present study demonstrated that Cyclin D1 expression also exhibited biphasic response to antiestrogens in these ER-negative breast cancer cells through the Src/EGFR/STAT5 pathway. The low concentrations of antiestrogens induced Src-mediated phosphorylation of the EGFR-Tyr-845 residue, which then recruits STAT5 as a downstream effector to induce Cyclin D1 expression through the GAS site located in the Cyclin D1 promoter. Src-dependent phosphorylation of EGFR-Tyr-845 is required for DNA synthesis induced by transactivating agonists of EGFR, such as endothelin, lysophosphatidic acid, cytokines and growth hormones [52]. It was reported that STAT5b, c-Src and EGFR play important roles in estrogen-stimulated proliferation of ER-positive breast cancer cells [30]. Introduction of dominant-negative STAT5a mutants into ER-positive T47D breast cancer cells inhibits estrogen-stimulated cell growth and induces apoptosis [31]. Thus, our results indicated that the EGFR/Src/STAT5 pathway is also involved in the biphasic antiestrogen signaling in ER-negative breast cancer cells.

In summary, we have shown that ER-α36 expressing ER-negative breast cancer cells exhibited biphasic response to antiestrogens, which further confirm that ER-α36 mediates non-genomic antiestrogen signaling. Our results also provided a possible explanation to the previous findings of the existence of two non-genomic estrogen-signaling pathways, one sensitive to antiestrogens and the other insensitive. The finding that antiestrogens at higher concentrations inhibit proliferation of ER-negative breast cancer cells through suppressing the EGFR/Src/STAT5 signaling pathway provided a rational for development of more effective therapeutic approaches for ER-negative breast cancer using combinations of antiestrogens with EGFR or Src inhibitors.

## Materials and Methods

### Chemicals and Antibodies

The Src inhibitors PP2, the PI3K inhibitor LY294002, 4-hydoxy tamoxifen (4-OHT) and ICI 182, 780 were from Tocris Bioscience (Ellisville, MO). The Src inhibitor dasatinib was obtained from LC Laboratories (Woburn, MA). Phospho-EGFR and -Src antibodies, EGFR and Src antibodies, anti-phospho-p44/42 ERK (Thr202/Tyr204) (197G2) mouse monoclonal antibody (mAb) and anti-p44/42 ERK (137F5) rabbit mAb were all purchased from Cell Signaling Technology (Boston, MA). Polyclonal anti-ER-α36 antibody was generated and characterized as described before [22]. Antibody for Cyclin D1 was purchased from Santa Cruz Biotechnology (Santa Cruz, CA).

### Cell Culture, Treatment and Growth Assay

MDA-MB-231 and MDA-MB-436 cells were obtained from American Type Culture Collection (ATCC, Manassas, VA). All parental and derivative cells were maintained at 37°C in a 10% CO_2_ atmosphere in DMEM and 10% fetal calf serum in a humidified incubator. For antiestrogen treatment, cells were maintained in phenol red-free media with 2.5% charcoal-stripped fetal calf serum for two to three days, and then in serum-free medium for 24 hours before experimentation. For ERK activation assays, cells were treated with vehicle (ethanol) and indicated concentrations of 4-OHT and ICI 182, 780. To test the effects of different inhibitors, all inhibitors were added 10 min. before addition of antiestrogens.

Since these ER-negative breast cancer cells express high levels of EGFR, which make cells proliferate at a near-maximal rate in serum-supplemented medium, the effects of antiestrogen signaling on proliferation of these cells are too subtle to detect most time. To alleviate this problem, we reduced charcoal-stripped fetal calf serum concentration in culture medium from 10% to 2.5% and increased estrogen treatment time to 12 days in our cell growth assays.

To examine cell growth in the presence or absence of antiestrogens, cells maintained for three days in phenol red-free DMEM plus 2.5% dextran-charcoal-stripped fetal calf serum (HyClone, Logan, UT) were treated with different concentrations of 4-OHT, ICI 182, 780 or ethanol vehicle as a control. The cells were seeded at 1×10^4^ cells per dish in 60 mm dishes and the cell numbers were determined using the ADAM automatic cell counter (Digital Bio., Korea) after 12 days. Five dishes were used for each treatment and experiments were repeated more than three times.

Cell lines with ER-α36 expression knocked down by the shRNA method in MDA-MB-231 and MDA-MB-436 cells were generated and described before [22].

### Plasmids, DNA transfection and Luciferase Assay

The expression vectors for a dominant-negative mutant of Src (pCMV5/SrcK295) and a constitutively active mutant of Src (pCMV5/SrcY527F) were obtained from Dr. Yun Qiu at the Department of Pharmacology and Experimental Therapeutics, University of Maryland School of Medicine. Dr. Linda Schuler at Department of Comparative Biosciences, University of Wisconsin-Madison kindly provided the luciferase reporter plasmids of the Cyclin D1 promoter (pl-963) carrying GAS1 and 2 mutations. Two dominant-negative STAT5 mutants, Stat5aΔ713 and Stat5aΔ740 were provided by Dr. H Yamashita at Department of Surgery II, Nagoya City University. The wild-type luciferase reporter plasmid of the Cyclin D1 promoter, Cyclin D1 pl-963 was obtained from Dr. Chris Albanese at Departments of Oncology and Pathology, Georgetown University Medical Center. The 4XM67 pTATA-TK-luciferase reporter plasmid was purchased from Addgene (Cambridge, MA). Cells were co-transfected with a cytomegalovirus-driven *Renilla* luciferase plasmid, pRL-CMV (Promega, Madison, WI) to establish transfection efficiency. Twenty-four hours after transfection, cells were treated with vehicle, 10 µM of dasatinib, PP2, or LY294002 for twenty-four hours. Forty-eight hours after transfection, cell extracts were prepared and luciferase activities were determined and normalized using the Dual-Luciferase Assay System (Promega, Madison, WI) and a TD 20/20 Luminometer (Turner BioSystems, Inc., Sunnyvale, CA) as instructed by the manufacturer.

### Western blot Analysis

For Western blot analysis, cells washed with cold PBS were lysed with the lysis buffer (50 mM Tris-HCl pH 8.0, 150 mM NaCl, 0.25 mM EDTA pH 8.0, 0.1% SDS, 1% Triton X-100, 50 mM NaF) supplemented with protease and phosphatase inhibitors from Sigma. The protein amounts were measured using the DC protein assay kit (BIO-RAD Laboratories, Hercules, CA). The same amounts of the cell lysates were boiled for 5 minutes in loading buffer and separated on a SDS-PAGE gel. After electrophoresis, the proteins were transferred to a PVDF membrane. The membranes were probed with various primary antibodies, HRP-conjugated secondary antibodies, and visualized with enhanced chemiluminescence (ECL) detection reagents (GE Healthcare Bio-Sciences Corp. Piscataway, NJ).

### Immunoprecipitation and Immunoblot Analysis

For imunoprecipitation assays, cells were washed twice with ice-cold PBS and lysed with the lysis buffer (150 mM NaCl, 20 mM TrisHCl, pH 7.4, 0.1% NP-40) supplemented with protease and phosphatase inhibitors (Sigma, St. Louis, MO). Cell lysates were then incubated with indicated anti-HA antibodies, or pre-immune serum and immunoprecipitated with protein A/G plus agarose. The precipitates were then washed, separated on SDS-PAGE and analyzed with Western blot analysis as described before [27], [29].

### Statistical analysis

Data were summarized as the mean ± standard error (SE) using the GraphPad InStat software program (GraphPad Software, La Jolla, CA, USA). Tukey-Kramer Multiple Comparisons Test was also used, and the significance was accepted for *P*<0.05.

## References

[1] Nilsson S, Makela S, Treuter E, Tujague M, Thomsen J (2001). Mechanisms of estrogen action.. Physiol Rev.

[2] Kelly MJ, Levin ER (2001). Rapid actions of plasma membrane estrogen receptors.. Trends Endocrinol Metab.

[3] Segars JH, Driggers PH (2002). Estrogen action and cytoplasmic signaling cascades. Part I: membrane-associated signaling complexes.. Trends Endocrinol Metab.

[4] Osborne CK, Coronado E, Allred DC, Wiebe V, DeGregorio M (1991). Acquired tamoxifen resistance: correlation with reduced breast tumor levels of tamoxifen and isomerization of trans-4-hydroxytamoxifen.. J Natl Cancer Inst.

[5] Osborne CK, Wiebe VJ, McGuire WL, Ciocca DR, DeGregorio MW (1992). Tamoxifen and the isomers of 4-hydroxytamoxifen in tamoxifen-resistant tumors from breast cancer patients.. J Clin Oncol.

[6] Howell A, Dodwell DJ, Anderson H (1990). New endocrine approaches to breast cancer.. Baillieres Clin Endocrinol Metab.

[7] Wakeling AE, Dukes M, Bowler J (1991). A potent specific pure antiestrogen with clinical potential.. Cancer Res.

[8] Shang Y, Hu X, DiRenzo J, Lazar MA, Brown M (2000). Cofactor dynamics and sufficiency in estrogen receptor-regulated transcription.. Cell.

[9] Fawell SE, White R, Hoare S, Sydenham M, Page M (1990). Inhibition of estrogen receptor-DNA binding by the “pure” antiestrogen ICI 164,384 appears to be mediated by impaired receptor dimerization.. Proc Natl Acad Sci U S A.

[10] Dauvois S, Danielian PS, White R, Parker MG (1992). Antiestrogen ICI 164,384 reduces cellular estrogen receptor content by increasing its turnover.. Proc Natl Acad Sci U S A.

[11] Nicholson RI, Gee JM, Manning DL, Wakeling AE, Montano MM (1995). Responses to pure antiestrogens (ICI 164384, ICI 182780) in estrogen-sensitive and -resistant experimental and clinical breast cancer.. Ann N Y Acad Sci.

[12] Zhao L, O'Neill K, Brinton RD (2006). Estrogenic agonist activity of ICI 182,780 (Faslodex) in hippocampal neurons: implications for basic science understanding of estrogen signaling and development of estrogen modulators with a dual therapeutic profile.. J Pharmacol Exp Ther.

[13] Sibonga JD, Dobnig H, Harden RM, Turner RT (1998). Effect of the high-affinity estrogen receptor ligand ICI 182,780 on the rat tibia.. Endocrinology.

[14] Wu J, Liang Y, Nawaz Z, Hyder SM (2005). Complex agonist-like properties of ICI 182,780 (Faslodex) in human breast cancer cells that predominantly express progesterone receptor-B: implications for treatment resistance.. Int J Oncol.

[15] Robertson JA, Zhang Y, Ing NH (2001). ICI 182,780 acts as a partial agonist and antagonist of estradiol effects in specific cells of the sheep uterus.. J Steroid Biochem Mol Biol.

[16] Dudley MW, Sheeler CQ, Wang H, Khan S (2000). Activation of the human estrogen receptor by the antiestrogens ICI 182,780 and tamoxifen in yeast genetic systems: implications for their mechanism of action.. Proc Natl Acad Sci U S A.

[17] Brinton RD (2001). Cellular and molecular mechanisms of estrogen regulation of memory function and neuroprotection against Alzheimer's disease: recent insights and remaining challenges.. Learn Mem.

[18] McEwen B (2002). Estrogen actions throughout the brain.. Recent Prog Horm Res.

[19] Zhao L, O'Neill K, Diaz Brinton R (2005). Selective estrogen receptor modulators (SERMs) for the brain: current status and remaining challenges for developing NeuroSERMs.. Brain Res Brain Res Rev.

[20] Wong JK, Le HH, Zsarnovszky A, Belcher SM (2003). Estrogens and ICI182,780 (Faslodex) modulate mitosis and cell death in immature cerebellar neurons via rapid activation of p44/p42 mitogen-activated protein kinase.. J Neurosci.

[21] Wang Z, Zhang X, Shen P, Loggie BW, Chang Y (2005). Identification, cloning, and expression of human estrogen receptor-alpha36, a novel variant of human estrogen receptor-alpha66.. Biochem Biophys Res Commun.

[22] Wang Z, Zhang X, Shen P, Loggie BW, Chang Y (2006). A variant of estrogen receptor-{alpha}, hER-{alpha}36: transduction of estrogen- and antiestrogen-dependent membrane-initiated mitogenic signaling.. Proc Natl Acad Sci U S A.

[23] Zou Y, Ding L, Coleman M, Wang Z (2009). Estrogen receptor-alpha (ER-alpha) suppresses expression of its variant ER-alpha 36.. FEBS Lett.

[24] Lee LM, Cao J, Deng H, Chen P, Gatalica Z (2008). ER-alpha36, a novel variant of ER-alpha, is expressed in ER-positive and -negative human breast carcinomas.. Anticancer Res.

[25] Shi L, Dong B, Li Z, Lu Y, Ouyang T (2009). Expression of ER-{alpha}36, a novel variant of estrogen receptor {alpha}, and resistance to tamoxifen treatment in breast cancer.. J Clin Oncol.

[26] Vranic S, Gatalica Z, Deng H, Frkovic-Grazio S, Lee LM (2011). ER-alpha36, a novel isoform of ER-alpha66, is commonly over-expressed in apocrine and adenoid cystic carcinomas of the breast.. J Clin Pathol.

[27] Kang L, Zhang X, Xie Y, Tu Y, Wang D (2010). Involvement of estrogen receptor variant ER-alpha36, not GPR30, in nongenomic estrogen signaling.. Mol Endocrinol.

[28] Lin SL, Yan LY, Zhang XT, Yuan J, Li M (2010). ER-alpha36, a variant of ER-alpha, promotes tamoxifen agonist action in endometrial cancer cells via the MAPK/ERK and PI3K/Akt pathways.. PLoS One.

[29] Zhang XT, Kang LG, Ding L, Vranic S, Gatalica Z (2011). A positive feedback loop of ER-alpha36/EGFR promotes malignant growth of ER-negative breast cancer cells.. Oncogene.

[30] Fox EM, Bernaciak TM, Wen J, Weaver AM, Shupnik MA (2008). Signal transducer and activator of transcription 5b, c-Src, and epidermal growth factor receptor signaling play integral roles in estrogen-stimulated proliferation of estrogen receptor-positive breast cancer cells.. Mol Endocrinol.

[31] Yamashita H, Iwase H, Toyama T, Fujii Y (2003). Naturally occurring dominant-negative Stat5 suppresses transcriptional activity of estrogen receptors and induces apoptosis in T47D breast cancer cells.. Oncogene.

[32] Brockman JL, Schroeder MD, Schuler LA (2002). PRL activates the cyclin D1 promoter via the Jak2/Stat pathway.. Mol Endocrinol.

[33] Zhang CC, Shapiro DJ (2000). Activation of the p38 mitogen-activated protein kinase pathway by estrogen or by 4-hydroxytamoxifen is coupled to estrogen receptor-induced apoptosis.. J Biol Chem.

[34] Mandlekar S, Yu R, Tan TH, Kong AN (2000). Activation of caspase-3 and c-Jun NH2-terminal kinase-1 signaling pathways in tamoxifen-induced apoptosis of human breast cancer cells.. Cancer Res.

[35] Obrero M, Yu DV, Shapiro DJ (2002). Estrogen receptor-dependent and estrogen receptor-independent pathways for tamoxifen and 4-hydroxytamoxifen-induced programmed cell death.. J Biol Chem.

[36] Mandlekar S, Kong AN (2001). Mechanisms of tamoxifen-induced apoptosis.. Apoptosis.

[37] Clarke R, Liu MC, Bouker KB, Gu Z, Lee RY (2003). Antiestrogen resistance in breast cancer and the role of estrogen receptor signaling.. Oncogene.

[38] Kang L, Wang ZY (2010). Breast cancer cell growth inhibition by phenethyl isothiocyanate is associated with down-regulation of oestrogen receptor-alpha36.. J Cell Mol Med.

[39] Mahfoudi A, Roulet E, Dauvois S, Parker MG, Wahli W (1995). Specific mutations in the estrogen receptor change the properties of antiestrogens to full agonists.. Proc Natl Acad Sci U S A.

[40] Pearce ST, Liu H, Jordan VC (2003). Modulation of estrogen receptor alpha function and stability by tamoxifen and a critical amino acid (Asp-538) in helix 12.. J Biol Chem.

[41] Nichols M, Rientjes JM, Stewart AF (1998). Different positioning of the ligand-binding domain helix 12 and the F domain of the estrogen receptor accounts for functional differences between agonists and antagonists.. EMBO J.

[42] Kurebayashi J, Otsuki T, Yamamoto S, Kurosumi M, Nakata T (1998). A pure antiestrogen, ICI 182,780, stimulates the growth of tamoxifen-resistant KPL-1 human breast cancer cells in vivo but not in vitro.. Oncology.

[43] Lubahn DB, Moyer JS, Golding TS, Couse JF, Korach KS (1993). Alteration of reproductive function but not prenatal sexual development after insertional disruption of the mouse estrogen receptor gene.. Proc Natl Acad Sci U S A.

[44] Gu Q, Korach KS, Moss RL (1999). Rapid action of 17beta-estradiol on kainate-induced currents in hippocampal neurons lacking intracellular estrogen receptors.. Endocrinology.

[45] Das SK, Taylor JA, Korach KS, Paria BC, Dey SK (1997). Estrogenic responses in estrogen receptor-alpha deficient mice reveal a distinct estrogen signaling pathway.. Proc Natl Acad Sci U S A.

[46] Pendaries C, Darblade B, Rochaix P, Krust A, Chambon P (2002). The AF-1 activation-function of ERalpha may be dispensable to mediate the effect of estradiol on endothelial NO production in mice.. Proc Natl Acad Sci U S A.

[47] Nethrapalli IS, Singh M, Guan X, Guo Q, Lubahn DB (2001). Estradiol (E2) elicits SRC phosphorylation in the mouse neocortex: the initial event in E2 activation of the MAPK cascade?. Endocrinology.

[48] Song RX, Barnes CJ, Zhang Z, Bao Y, Kumar R (2004). The role of Shc and insulin-like growth factor 1 receptor in mediating the translocation of estrogen receptor alpha to the plasma membrane.. Proc Natl Acad Sci U S A.

[49] Migliaccio A, Castoria G, Di Domenico M, de Falco A, Bilancio A (2000). Steroid-induced androgen receptor-oestradiol receptor beta-Src complex triggers prostate cancer cell proliferation.. EMBO J.

[50] Cowell LN, Graham JD, Bouton AH, Clarke CL, O'Neill GM (2006). Tamoxifen treatment promotes phosphorylation of the adhesion molecules, p130Cas/BCAR1, FAK and Src, via an adhesion-dependent pathway.. Oncogene.

[51] Superti-Furga G, Courtneidge SA (1995). Structure-function relationships in Src family and related protein tyrosine kinases.. Bioessays.

[52] Zwick E, Hackel PO, Prenzel N, Ullrich A (1999). The EGF receptor as central transducer of heterologous signalling systems.. Trends Pharmacol Sci.

